# Chalcomycins from Marine-Derived *Streptomyces* sp. and Their Antimicrobial Activities

**DOI:** 10.3390/md15060153

**Published:** 2017-05-29

**Authors:** Shutai Jiang, Lili Zhang, Xuechang Pei, Fang Deng, Dan Hu, Guodong Chen, Chuanxi Wang, Kui Hong, Xinsheng Yao, Hao Gao

**Affiliations:** 1 Institute of Traditional Chinese Medicine and Natural Products, College of Pharmacy/Guangdong Province Key Laboratory of Pharmacodynamic Constituents of Traditional Chinese Medicine & New Drug Research, Jinan University, Guangzhou 510632, China; vstjiang@stu2014.jnu.edu.cn (S.J.); qymuzinini@126.com (L.Z.); cyan2014@stu2014.jnu.edu.cn (X.P.); xff@stu2014.jnu.edu.cn (F.D.); thudan@jnu.edu.cn (D.H.); chgdtong@jnu.edu.cn (G.C.); tyaoxs@jnu.edu.cn (X.Y.); 2 Key Laboratory of Combinatorial Biosynthesis and Drug Discovery, Ministry of Education, and School of Pharmaceutical Sciences, Wuhan University, Wuhan 430071, China; kuihong31@whu.edu.cn; 3 Food and Drug Department, Qingyuan Polytechnic, Qingyuan 511510, China

**Keywords:** marine-derived *Streptomyces*, secondary metabolite, 16-membered macrolide, chalcomycin E, antimicrobial activity

## Abstract

Dihydrochalcomycin (**1**) and chalcomycin (**2**), two known chalcomycins, and chalcomycin E (**3**), a new compound, were isolated from marine-derived *Streptomyces* sp. HK-2006-1. Their structures were elucidated by detailed spectroscopic and X-ray crystallographic analysis. The antimicrobial activities against *Staphylococcus aureus*, *Escherichia coli*, *Candida albicans*, and *Aspergillus niger* of **1**–**3** were evaluated. Compounds **1**–**2** exhibited activities against *S. aureus* with minimal inhibitory concentrations (MICs) of 32 µg/mL and 4 µg/mL, respectively. The fact that **1**–**2** showed stronger activity against *S. aureus* 209P than **3** indicated that the epoxy unit was important for antimicrobial activity. This structure–activity tendency of chalcomycins against *S. aureus* is different from that of aldgamycins reported in our previous research, which provide a valuable example for the phenomenon that 16-membered macrolides with different sugars do not have parallel structure–activity relationships.

## 1. Introduction

Infectious diseases seriously imperil human health. Antibiotics are important medicines against infectious diseases [1]. However, the prolonged, extensive, and indiscriminate use of antibiotics has triggered widespread resistance [2]. The global epidemic of continually rising resistance has become a critical threat to human health and therefore the discovery of new antibiotics is urgently needed [2]. Macrolide antibiotics such as erythromycins, tylosins, avermectins, and milbemycins have significant activity against a broad spectrum of Gram-positive bacteria [3,4,5], playing an important role in the chemotherapy of infectious diseases [6,7]. Macrolides are usually characterized by a 12-, 14-, 16-, 18-, 20-, 22-, or 24-membered lactone ring with one or more sugar moieties [3,8]. Different types of macrolides have different structure-antimicrobial activity relationships. For example, 16-membered macrolides with different sugars have no parallel structure–activity tendencies. Omura reported that the structure–activity relationships of some 16-membered macrolides (rosamicins, angolamycins, and neutramycins) differed from the evidence found in other 16-membered macrolides (leucomycins) [9]. The 16-membered macrolides with different sugar moiety for instance spiramycins, neospiramycins, and forocidins have different structure–activity relationships [10].

**Figure 1:**
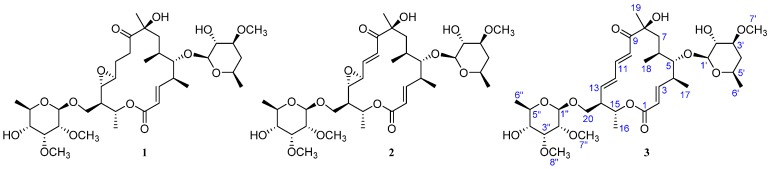
The structures of compounds **1**–**3**.

Many interesting strains were obtained in our continuing investigations on active components from microorganisms. Among our recent discoveries [11,12,13,14,15,16,17,18,19,20,21], we recently reported that a strain of *Streptomyces* sp. HK-2006-1 produced both aldgamycins and chalcomycins, which are 16-membered macrolides [11,21]. Chalcomycin and seven aldgamycins were isolated from this strain, and chalcomycin showed more potent antibacteria activity against *Staphylococcus aureus* than aldgamycins [11]. Chalcomycin, the first member of chalcomycins, was reported with its activity against bacteria as early as 1962 [22]. However, there have only been seven chalcomycins (chalcomycin, chalcomycins B-D, dihydrochalcomycin, 8-deoxy-chalcomycin, 250-144C) reported until now [23,24,25,26,27], and there is no discussion on the structure-antimicrobial activity relationship of chalcomycins against *S. aureus*. Thus, in this study, the fermentation volume of this strain *Streptomyces* sp. HK-2006-1 was scaled up in search of more chalcomycins. The crude extract of the culture of the strain was subjected to column chromatography (CC) over silica gel, Sephadex LH-20, octadecylsilane (ODS), and high performance liquid chromatography (HPLC), yielding three chalcomycins, dihydrochalcomycin (**1**), chalcomycin (**2**), and a new compound, chalcomycin E (**3**) (Figure 1). In addition, their antimicrobial activities against two bacteria, Gram-positive *S. aureus* 209P and Gram-negative *Escherichia coli* ATCC0111, as well as two fungi, *Candida albicans* FIM709 and *Aspergillus niger* R330, were evaluated. Details of the isolation, structural elucidation, and antimicrobial activities of compounds **1**–**3** are presented herein.

## 2. Results and Discussion

**Figure 2:**
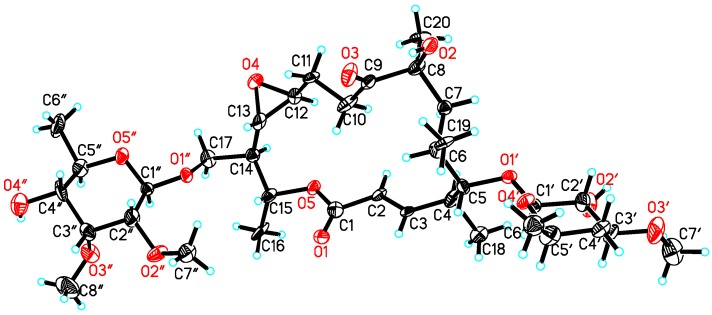
X-ray structure of **1**.

Compounds **1** and **2** were established as dihydrochalcomycin and chalcomycin respectively by precisely comparing the nuclear magnetic resonance (NMR) data with literature values [11,24,28]. The single-crystal X-ray crystallographic analysis of dihydrochalcomycin (**1**) was reported for the first time (Figure 2). Chalcomycin (**2**) was also obtained and identified in our previous study on the strain of *Streptomyces* sp. HK-2006-1 [11].

**Figure 3:**
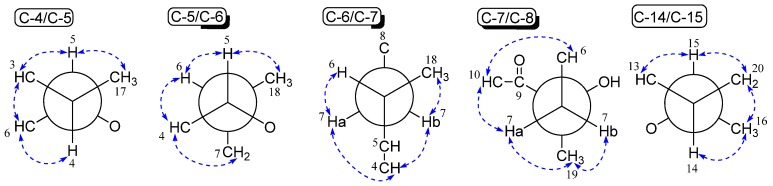
The observed rotating frame overhauser effect spectroscopy (ROESY) correlations (dashed double arrow in blue) of C-4−C-5−C-6−C-7−C-8 and C-14−C-15 in **3**.

Compound **3** was obtained as a white amorphous powder. The quasi-molecular ion at *m/z* 707.3616 [M + Na]^+^ by high resolution electrospray ionization mass spectroscopy (HRESIMS) indicated that the molecular formula of **3** was C_35_H_56_O_13_ (eight degrees of unsaturation), which was 16 atomic mass unit (O) less than **2**. Analysis of its ^1^H and ^13^C NMR spectroscopic data (Table 1) revealed nearly identical structure features to **2**, except that two mono-oxygenated methine carbons at δ_C_ 59.0 and 58.7 disappeared, and two olefinic carbons at δ_C_ 143.3 and 133.0 appeared. Analysis of ^1^H−^1^H COSY and the coupling values of the protons revealed the presence of the spin system C-10−C-11−C-12−C-13−C-14(C-20)−C-15−C-16. Therefore, **3** was the reduction product of **2** at C-12/C-13. The geometrical configuration of the double bond moiety (C-12/C-13) was deduced as *E* on the basis of the coupling constant of the olefinic protons (*J*_12,13_ = 14.1 Hz). Thus, compound **3** can be recognized as a new member of the chalcomycin family, consisting of the 16-membered lactone ring, mycinose, and chalcose, and its structure was further confirmed by two-dimensional NMR (2D NMR) data (Table 1 and Table S1). The observed rotating frame overhauser effect spectroscopy (ROESY) correlations (Figure 3) were consistent with the stereochemistry of the 16-membered lactone ring. All the reported mycinose and chalcose units in natural products have D configurations. The mycinose and chalcose units in the isolated macrolides from the strain of *Streptomycetes* sp. HK-2006-1 also had D configurations [11]. Therefore, the absolute configurations of the mycinose and chalcose units in **3** were assumed to be D. The relative configurations of the two units were established as β from the coupling constants of the anomeric protons (H-1′ and H-1′′). Thus, the structure of **3** was elucidated as (3*E*,5*S*,6*S*,7*S*,9*S*,11*E*,13*E*,15*R*,16*R*)-9-hydroxy-15-(((2*R*,3*R*,4*R*,5*R*,6*R*)-5-hydroxy-3,4-dimethoxy-6-methyltetrahydro-2*H*-pyran-2-yloxy)methyl)-6-((2*S*,3*R*,4*S*,6*R*)-3-hydroxy-4-methoxy-6-methyltetrahydro-2*H*-pyran-2-yl)oxy)-5,7,9,16-tetramethyloxacyclohexadeca-3,11,13-triene-2,10-dione, and named as chalcomycin E.

Until now, only seven chalcomycins had been reported. The discovery of chalcomycin E (**3**) adds a new member to chalcomycins. The single-crystal X-ray crystallographic analysis of dihydrochalcomycin (**1**) was firstly reported. Compounds **1**–**3** were tested for antimicrobial activities against two bacteria, Gram-positive *S. aureus* 209P and Gram-negative *E. coli* ATCC0111, as well as two fungi, *C. albicans* FIM709 and *A. niger* R330 (Table 2). Compounds **1**–**2** showed activities against *S. aureus*, but no activity against the other test strains. The fact that **1** and **2** exhibited stronger activity against *S. aureus* 209P than **3** suggested that the epoxy unit was important for antimicrobial activity. However, the replacement of the double bond in C-10 to C-13 by the epoxy unit in aldgamycins is not beneficial for antimicrobial activity. The difference in structure between aldgamycins and chalcomycins is just the sugar type at C-5, but the two types of macrolides have different structure–activity tendencies. Our findings provide a valuable example for the phenomenon that 16-membered macrolide antibiotics with different sugars do not have parallel structure–activity relationships [9,10].

## 3. Conclusions

Two known chalcomycins, dihydrochalcomycin (**1**) and chalcomycin (**2**), together with a new one, chalcomycin E (**3**) were isolated from marine-derived *Streptomyces* sp. HK-2006-1. Their structures were determined by detailed spectroscopic and X-ray crystallographic analysis. The discovery of chalcomycin E (**3**) adds a new member to chalcomycins. The antimicrobial activities of **1**–**3** were tested against *S. aureus*, *E. coli*, *C. albicans*, and *A. niger*. Compounds **1**–**2** showed activities against *S. aureus* with minimal inhibitory concentrations (MICs) of 32 µg/mL and 4 µg/mL, respectively. Compounds **1**–**2** showed stronger activity against *S. aureus* 209P than **3**, which suggested a different structure–activity tendency against *S. aureus* from that of aldgamycins. This case indicated that 16-membered macrolide antibiotics with different sugars do not have parallel structure–activity relationships.

## Figures and Tables

**Table 1:** NMR (600 MHz, CDCl_3_) data for **3**.

Position	*δ*_C_, Mult.	*δ*_H_ (*J* in Hz) ^§^	^1^H, ^1^H-COSY	HMBC	ROESY
aglycone					
1	165.6, C	–	–	–	–
2	121.4, CH	5.75 d (15.4)	3	4	4, 17
3	151.7, CH	6.62 dd (15.4, 9.5)	2, 4	1	5, 6, 17
4	41.0, CH	2.66	3, 5, 17	2, 3	2, 6, 7a, 7b
5	88.1, CH	3.19	4, 6	3, 4, 6, 7, 17, 18, 1′	3, 6, 17, 18, 1′
6	34.0, CH	1.30	5, 7a, 7b, 18	–	3, 4, 5, 7a, 10
7	37.4, CH_2_	1.89, Ha	6, 7b	6, 8, 9, 18	4, 6, 10, 19
		1.83, Hb	6, 7a	6, 18	4, 18, 19
8	78.3, C	–	–	–	–
9	202.0, C	–	–	–	–
10	122.0, CH	6.18 d (15.1)	11	8, 9, 11, 12	6, 7a, 19
11	144.1, CH	7.30 dd (15.1, 10.1)	10, 12	9, 12, 13	–
12	133.0, CH	6.15 dd (14.1, 10.1)	11, 13	10, 11, 13, 14	–
13	143.3, CH	6.14 dd (14.1, 9.2)	12, 14	11, 12, 14, 20	15, 20b
14	51.2, CH	2.47	13, 15, 20a, 20b	12, 13, 15	16, 20a
15	69.2, CH	5.06 dq (10.2, 6.2)	14, 16	1, 13, 14	13, 20a, 20b
16	18.6, CH_3_	1.36 d (6.3)	15	14, 15	14, 20a, 20b
17	19.2, CH_3_	1.18 d (6.9)	4	3, 4, 5	2, ,3, 5, 1′
18	19.3, CH_3_	1.00 d (6.9)	6	5, 6, 7	5, 7b
19	27.9, CH_3_	1.38 s	–	7, 8, 9	7a, 7b, 10
20	68.4, CH_2_	4.04 dd (9.6, 3.7), Ha	14, 20b	13, 14, 15, 1″	14, 15, 16, 20b, 1″
		3.57 dd (9.6, 6.1), Hb	14, 20a	13, 14, 15, 1″	13, 15, 16, 20a, 1″
β-d-chalcose unit					
1′	103.0, CH	4.19 d (7.6)	2′	5, 5′	5, 17, 3′, 5′
2′	75.1, CH	3.32 dd (8.8, 7.6)	1′, 3′	1′, 3′, 4′	4′b
3′	80.4, CH	3.22	2′, 4′a, 4′b	1′, 2′, 4′, 7′	1′, 4′a, 5′
4′	36.8, CH_2_	2.04 ddd (12.7, 4.9, 1.9), Ha	3′, 4′b, 5′	2′, 3′	3′, 5′, 6′
		1.25, Hb	3′, 4′a, 5′	2′, 3′, 5′	2′, 6′
5′	67.8, CH	3.48	4′a, 4′b, 6′	1′	1′, 3′, 4′a
6′	20.9, CH_3_	1.23 d (6.2)	5′	4′, 5′	4′a, 4′b
7′	56.7, CH_3_	3.41 s	–	3′	–
β-d-mycinose unit					
1″	101.1, CH	4.58 d (7.8)	2″	20, 3″, 5″	20a, 20b, 5″, 8″
2″	81.9, CH	3.04 dd (7.8, 3.1)	1″, 3″	1″, 7″	3″, 4″, 7″
3″	79.8, CH	3.76 t (3.1)	2″, 4″	1″, 2″, 4″, 5″, 8″	2″, 4″, 8″
4″	72.7, CH	3.18	3″, 5″	2″	2″, 3″, 6″
5″	70.6, CH	3.52	4″, 6″	3″, 4″	1″
6″	17.8, CH_3_	1.27 d (6.2)	5″	4″, 5″	4″
7″	59.8, CH_3_	3.52 s	–	2″	2″
8″	61.8, CH_3_	3.62 s	–	3″	1″, 3″

^§^ Indiscernible signals owing to overlapping or having complex multiplicity are reported without designating multiplicity. NMR: nuclear magnetic resonance; ^1^H, ^1^H COSY: ^1^H, ^1^H chemical shift correlated spectroscopy; HMBC: heteronuclear multiple-bond correlation; ROESY: rotating frame overhauser effect spectroscopy.

**Table 2:** Antimicrobial activities of **1**–**3** (minimal inhibitory concentrations (MICs): µg/mL).

Compound	Bacteria		Fungi	
	*S. aureus*	*E. coli*	*C. albicans*	*A. niger*
**1**	32	>512	>512	>512
**2**	4	>512	>512	>512
**3**	>512	>512	>512	>512
Tobramycin	0.4	2	NT	NT
Actidione	NT	NT	64	32

NT: not tested.

## Supplementary Materials

The following are available online at www.mdpi.com/1660-3397/15/6/153/s1, materials and methods, one-dimensional NMR (1D NMR) data and spectra for **1** and **2**, and 1D/2D NMR, ultraviolet (UV), and HRESIMS spectra for **3**.
