# Supplementary material for: Chalcomycins from Marine-Derived Streptomyces sp. and Their Antimicrobial Activities
Source: Mar Drugs. 2017 May 29;15(6):153. doi: 10.3390/md15060153 (PMC5484103; doi:10.3390/md15060153)
Supplement: Supplementary file 1 [file marinedrugs-15-00153-s001.pdf]

Supporting Information

Chalcomycins from Marine-derived *Streptomyces* sp. and Their Antimicrobial Activities

Shutai Jiang[1,†], Lili Zhang[1,3,†], Xuechang Pei[1], Fang Deng[1], Dan Hu[1], Guodong Chen[1], Chuanxi Wang[1,*], Kui Hong[2], Xinsheng Yao[1], and Hao Gao[1,*]

[1] Institute of Traditional Chinese Medicine and Natural Products, College of Pharmacy / Guangdong Province Key Laboratory of Pharmacodynamic Constituents of Traditional Chinese Medicine & New Drug Research, Jinan University, Guangzhou 510632, People's Republic of China; vstjiang@stu2014.jnu.edu.cn (S.J.); qymuzinini@126.com (L.Z.); cyan2014@stu2014.jnu.edu.cn (X.P.); xff@stu2014.jnu.edu.cn (F.D.); thudan@jnu.edu.cn (D.H.); chgdtong@jnu.edu.cn (G.C.); tyaoxs@jnu.edu.cn (X.Y.)

[2] Key Laboratory of Combinatorial Biosynthesis and Drug Discovery (Wuhan University), Ministry of Education, and Wuhan University School of Pharmaceutical Sciences, Wuhan, 430071, People's Republic of China; kuihong31@whu.edu.cn (K.H.)

[3] Food and Drug Department, Qingyuan Polytechnic, Qingyuan 511510, People's Republic of China; qymuzinini@126.com (L.Z.)

***** Correspondence: tghao@jnu.edu.cn (H.G.); tcxwang@jnu.edu.cn (C.W.); Tel./Fax: +86-20-8522-8369 (H.G. & C.W.)

[†] These authors have contributed equally to this work.

List of Supplementary Information

1. Materials and methods 2
    1.1 General experimental procedure 2
    1.2 Bacterial material 2
    1.3 Fermentation, extraction, and isolation 3
    1.4 X-ray crystallographic analysis of **1** 4
    1.5 Antimicrobial assay 5
2. The NMR data for **1** and **2**. 6
3. The 1D NMR spectra for dihydrochalcomycin (**1**) 7
4. The 1D NMR spectra for chalcomycin (**2**) 8
5. The 1D and 2D NMR spectra for chalcomycin E (**3**) 10
6. The UV spectrum for chalcomycin E (**3**) 13
7. The HRESIMS spectrum for chalcomycin E (**3**) 14
8. References 14

1. Materials and methods

1.1 General experimental procedure

Optical rotations were measured on a JASCO P1020 digital polarimeter (Jasco International, Tokyo, Japan). UV data were recorded on a JASCO V-550 UV/ vis spectrometer (Jasco International, Tokyo, Japan). IR data were recorded using a JASCO FT/IR-480 plus spectrometer (Jasco International, Tokyo, Japan). ^{1}H and ^{13}C NMR spectra were acquired with Bruker AV 400 (**1** and **2**) and Bruker AV 600 (**3**) (Bruker BioSpin Group, Faellanden, Switzerland) using solvent signals ($CDCl_3$: δ_H 7.26/δ_C 77.0) as internal standard. The ESIMS spectra were performed on a Bruker amazon SL mass spectrometer (Bruker Daltonics Int., Boston, USA) and the HRESIMS spectra were obtained on Waters Synapt G2 mass spectrometer (Waters Corporation, Milford, MA, USA). The analytical HPLC was performed on a Dionex HPLC system (Thermo Fisher Scientific, Inc., Waltham, MA, USA) using a YMC-Pack ODS-A column (4.6 × 250 mm, 5 μm) (YMC, Tokyo, Japan). Semi-preparative HPLC was performed on a Dionex HPLC system (Thermo Fisher Scientific, Inc., Waltham, MA, USA) using a YMC-Pack ODS-A column (10.0 × 250 mm, 5 μm) (YMC, Tokyo, Japan). The medium-pressure liquid chromatography (MPLC) apparatus was equipped with a dual pump gradient system, a UV preparative detector, and a Dr Flash II fraction collector system (Shanghai Lisui E-Tech, Shanghai, People's Republic of China). Column chromatography (CC) was performed on silica gel (200–300 mesh) (Qingdao Haiyang Chemical, Qingdao, People's Republic of China), DIAION HP-20 (Mitsubishi Chemical, Tokyo, Japan), ODS (50 μm, YMC, Tokyo, Japan), and Sephadex LH-20 (Amersham Pharmacia Biotech, Atlanta, GA, USA). Methanol, ethyl acetate and trichloromethane (analytical grade) were purchased from Fuyu Fine Chemical Co., Ltd. (Tianjin, China). Methanol (HPLC grade) was purchased from Yuwang Industrial Co., Ltd. (Yucheng, China). Acetonitrile (HPLC grade) was purchased from Oceanpak Alexative Chemical Co., Ltd. (Gothenburg, Sweden). Tobramycin was purchased from Aladdin (Shanghai, China). Actidione was purchased from Biosharp (Hefei, China).

1.2 Bacterial material

The strain numbered HK-2006-1 was isolated from marine sediment by one of the authors (K. H.). The strain was identified as *Streptomyces* sp. based on the

morphological characteristics and gene sequence analysis. The 16s rRNA gene sequence of the strain was deposited at GenBank as KF982843.1. The strain was deposited in Institute of Traditional Chinese Medicine and Natural Products, College of Pharmacy, Jinan University, Guangzhou, China, with the access code, HK-2006-1.

1.3 Fermentation, extraction, and isolation

The strain was grown on modified YE agar slant culture-medium, consisting of 0.4% yeast extract, 1.0% malt flour, 0.4% glucose, 1.8% marine salt and 2.0% agar in the distilled water (pH 7.4). After 7 days of inoculation at 28 °C, a mycelium was inoculated into each of the 500 mL Erlenmeyer flasks which contained 60 mL of liquid modified FM3 medium, consisting of 2.0% starch, 0.5% yeast extract, 1.5% soybean powder, 2.0% peptone, 0.4% $CaCO_3$, and 1.8% marine salt in the distilled water (pH 7.0). The flasks were incubated at 28 °C on a rotary shaker (200 rpm) for 48 hours. Each seed culture (60 mL) was transferred into a 3000 mL Erlenmeyer flask containing 1000 mL of modified FM3 medium. The flasks were incubated on a rotary shaker (180 rpm) at 28 °C for 7 days. After fermentation, the culture (120 L) was centrifuged to yield supernatant and a mycelium cake. The supernatant was adsorbed onto a column of DIAION HP-20 packed with water (5.0 L). The column was eluted with ethanol (20 L), and then the eluent was concentrated in vacuo to give a crude material. The mycelium cake was extracted with 15 L of ethanol and the organic phase was evaporated to dryness. The two organic parts were combined and then dissolved in water (1 L). The solution was further extracted with EtOAc (1 L × 3) to afford an EtOAc fraction (13.8 g). The EtOAc fraction (13.8 g) was subjected to silica gel CC using a successive elution of chloroform – methanol (99:1, 95:5, 90:10; 80:20, 70:30, 60:40, 50:50, and 0:100, v/v) to afford 6 fractions (Fr.1–Fr.6) on the basis of TLC. Fraction 3 (7.2 g), which were the elution part of chloroform – methanol (90:10 and 80:20, v/v), was subjected to Sephadex LH-20 CC using MeOH to give 5 fractions (Fr.3–1 to Fr.3–5). The fraction Fr.3–2 (3.9 g) was further subjected to MPLC on ODS CC (4 × 40 cm), eluted with a MeOH–H_2O gradient (5%–100%, v/v) for 320 min with a flow rate of 10 mL/min to yield 13 fractions (Fr.3–1–1 to Fr.3–1–13). The fraction Fr.3–1–8 (198.2 mg) was purified on semi-preparative RP-HPLC using CH_3CN–H_2O (50:50, v/v) at a flow rate of 1.5 mL/min to yield **2** (t_R: 20.3 min, 11.2 mg). The fraction Fr.3–1–9 (170.6 mg) was purified on semi-preparative RP-HPLC using CH_3CN–H_2O (50:50, v/v) at a flow rate of 1.5

mL/min to yield **1** (t_R: 23.9 min, 16.3 mg). The fraction Fr.3–1–10 (160.7 mg) was purified on semi-preparative RP-HPLC using $CH_3CN–H_2O$ (55:45, v/v) at a flow rate of 1.5 mL/min to yield **3** (t_R: 22.5 min, 8.3 mg).

Chalcomycin E (**3**): white amorphous powder; $[\alpha]_D^{30}$ +8.3 (*c* 0.95, MeOH); UV (MeOH) λ_{max} (log *ε*) 204 (3.44), 216 (3.54), 284 (3.61) nm; IR (KBr) ν_{max} 3465, 2972, 2928, 2357, 2338, 1715, 1679 ,1649, 1595, 1454, 1377, 1343, 1170, 1079 cm^{-1}; ^1H ($CDCl_3$, 600 MHz) and ^{13}C NMR ($CDCl_3$, 150 MHz) see Table 1; ESIMS (positive) m/z 707.7 $[M + Na]^+$; HRESIMS (positive) *m/z* 707.3616 $[M + Na]^+$ (calcd. for $C_{35}H_{56}O_{13}Na$, 707.3619).

1.4 X-ray crystallographic analysis of 1

Upon crystallization from CH_3OH-H_2O using the vapor diffusion method, colorless needle crystals of **1** were obtained. Data were collected using a Sapphire CCD with graphite mono chromated Cu Kα radiation, λ = 1.54184 Å at 173.0 K. Crystal data: $C_{35}H_{58}O_{14}$, 6(H_2O), M = 810.91, space group monoclinic, P 21; unit cell dimensions were determined to be a = 13.932(3) Å, b = 25.605(5) Å, c = 14.049(3) Å, α = 90.00 °, β = 119.73(3) °, γ = 90.00 °, V = 4352.2(19) $Å^3$, Z = 4, Dx = 1.219 mg/m^3, F (000) = 1712, μ (Cu Kα) = 0.851 mm^{-1}. 47704 reflections were collected to θ_{max} = 62.766°, in which independent unique 12951 reflections (R_{int} = 0.0429, R_{sigma} = 0.0364) were used in all calculations. The structure was solved by direct methods using the SHELXS program, and refined by the program SHELXL and full-matrix least-squares calculations. In the structure refinements, nonhydrogen atoms were placed on the geometrically ideal positions by the "ride on" method. Hydrogen atoms bonded to oxygen were located by the structure factors with isotropic temperature factors. The final refinement gave R_1 = 0.1467 (I >2σ(I)), wR_2 = 0.3735 (I >2σ(I)), wR_1 = 0.1482 (all data), wR_2 = 0.3745 (all data), S = 1.066, and Flack = 0.5. Crystallographic data for dihydrochalcomycin (**1**) has been deposited in the Cambridge Crystallographic Data Center as supplementary publication No. CCDC 1025471. Copies of the data can be obtained, free of charge, on application to the Director, CCDC, 12 Union Road,

Cambridge CB2 1EZ, UK (fax: +44-(0)1223-336033, or e-mail: deposit@ccdc.cam.ac.uk).

1.5 Antimicrobial assay

The antimicrobial activities of Compounds **1-3** were tested by paper disk diffusion method [1, 2]. Seed cultures of two bacteria (*S. aureus* 209P, *E. coli* ATCC0111) and two fungi (*C. albicans* FIM709, A. niger R330) were prepared by incubating the organism at 32 °C (fungi) or 37 °C (bacteria), respectively, for 12 hours. Aliquots of the overnight cultures (80 µL) were lawned onto the surfaces of nutrient agar (bacteria) or Sabouraud's dextrose agar (fungi), respectively. Sterile filter disks (6 mm diameter) that were infused with 8 µL of test solution (in DMSO), positive control and vehicle only (DMSO) were added to the plates. The plates were left upright for 30 min at room temperature before being placed in an incubator at 32 °C (fungi) for 24 hours or 37 °C (bacteria) for 12 hours, respectively, and then the zones of growth inhibition around each disk were observed.

The continuous 2-fold dilution method was used to evaluate the minimal inhibitory concentrations (MICs) [3, 4]. Seed cultures of two bacteria (*S. aureus* 209P and *E. coli* ATCC0111) and two fungi (*C. albicans* FIM709 and *A. niger* R330) were prepared by incubating these microbes at 37 °C (bacteria) and 32 °C (fungi) for 12 hours in liquid beef extract medium and Sabouraud's medium, respectively. The above seed cultures were diluted to a concentration of 1.8 × 10^{10} CFU/mL and then added to each well of 96-well plate (100 µL/ well). These microbes were treated with the continuous 2-fold dilution of compounds (from 512 to 0.25 µg/mL). The 96-well plates were placed in an incubator at 32 °C (fungi) for 24 hours or 37 °C (bacteria) for 12 hours, respectively. The MICs were defined as the lowest concentration at which no microbial growth could be observed.

2. The NMR data for 1 and 2.

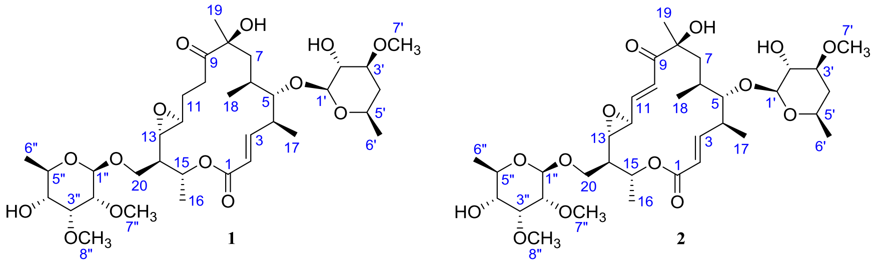

Table S1. NMR (400 MHz, $CDCl_3$) data for 1 and 2.

	1		2	
Position	δ_C, mult.	δ_H (*J* in Hz)§	δ_C, mult.	δ_H (*J* in Hz)§
	aglycone		aglycone	
1	165.6, C	-	165.3, C	-
2	121.2, CH	5.85 d (15.5)	120.8, CH	5.82 d (15.4)
3	151.4, CH	6.73 dd (15.5, 10.6)	151.6, CH	6.65 dd (15.4, 10.4)
4	41.8, CH	2.75	41.7, CH	2.70
5	87.4, CH	3.26 br.d (9.8)	87.8, CH	3.21
6	34.3, CH	1.30	34.0, CH	1.26
7	37.0, CH_2	1.90, Ha	37.0, CH_2	1.96, Ha
		1.88, Hb		1.90, Hb
8	79.6, C	-	78.4, C	-
9	212.7, C	-	200.1, C	-
10	32.6, CH_2	2.71, Ha	124.9, CH	6.58 d (15.5)
		2.14, Hb		
11	27.3, CH_2	2.00, Ha	146.4, CH	6.56 dd (15.5, 9.3)
		1.54, Hb		
12	59.3, CH	2.74	58.7, CH	3.31
13	58.0, CH	2.83 dd (9.0, 1.9)	59.0, CH	3.14 dd (9.2, 1.9)
14	48.6, CH	1.35	49.5, CH	1.38
15	69.8, CH	5.32 dq (10.7, 6.2)	68.7, CH	5.33 dq (10.8, 6.3)
16	18.5, CH_3	1.34 d (6.2)	18.6, CH_3	1.34 d (6.3)
17	18.3, CH_3	1.24 d (6.0)	18.3, CH_3	1.21 d (6.9)
18	18.8, CH_3	0.99 d (6.8)	19.2, CH_3	1.01 d (6.9)
19	28.1, CH_3	1.37 s	27.8, CH_3	1.38 s
20	67.2, CH_2	4.14 dd (10.0, 3.3), Ha	66.9, CH_2	4.18 dd (10.4, 3.3), Ha
		3.62 dd (10.0, 3.0), Hb		3.66 dd (10.1, 3.3), Hb
	β-D-chalcose		*β*-D-chalcose	
1'	103.2, CH	4.24 d (7.5)	103.2, CH	4.21 d (7.5)
2'	75.0, CH	3.33 dd (7.5, 3.0)	75.0, CH	3.32
3'	80.4, CH	3.23	80.5, CH	3.22
4'	36.8, CH_2	2.04, Ha	36.7, CH_2	2.04 ddd (12.7, 4.8, 1.8), Ha
		1.21, Hb		1.24, Hb
5'	67.8, CH	3.48	67.8, CH	3.47
6'	20.9, CH_3	1.22 d (6.2)	20.8, CH_3	1.22 d (6.2)
7'	56.7, CH_3	3.41 s	56.7, CH_3	3.41 s
	β-D-mycinose		*β*-D-mycinose	
1"	100.9, CH	4.56 d (7.7)	100.9, CH	4.57 d (7.8)
2"	81.9, CH	3.07 dd (7.7, 3.1)	82.0, CH	3.07 dd (7.8, 2.8)
3"	79.6, CH	3.76 t (3.1)	79.6, CH	3.76 t (3.1)
4"	72.7, CH	3.22	72.7, CH	3.19
5"	70.8, CH	3.53	70.7, CH	3.52
6"	17.8, CH_3	1.25 d (6.2)	17.7, CH_3	1.26 d (6.3)
7"	59.6, CH_3	3.55 s	59.6, CH_3	3.55 s
8"	61.7, CH_3	3.61 s	61.7, CH_3	3.62 s

§ Indiscernible signals owing to overlapping or having complex multiplicity are reported without designating multiplicity.

3. The 1D NMR spectra for dihydrochalcomycin (1)

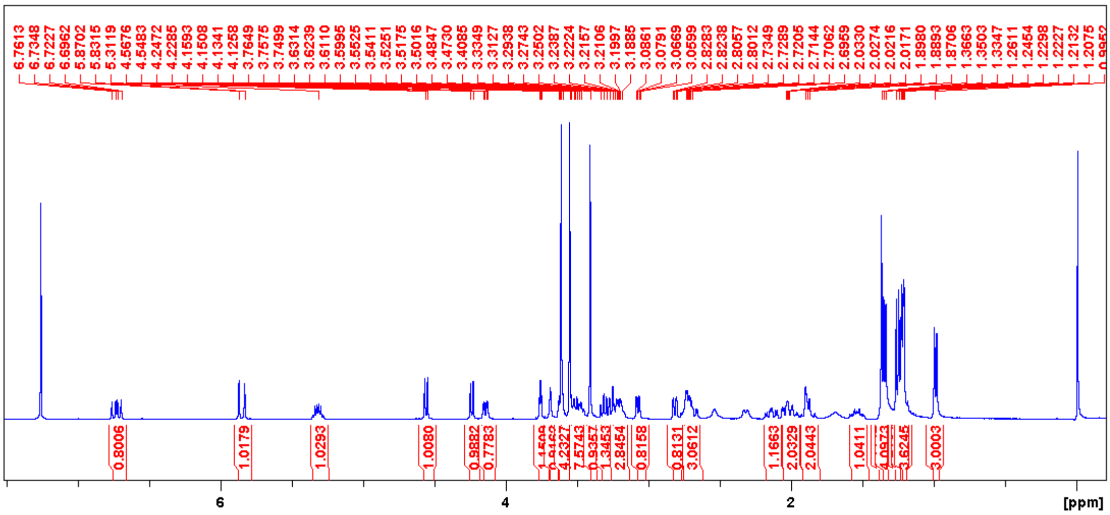

Figure S1. ^{1}H **NMR (400 MHz, $CDCl_3$) spectrum for dihydrochalcomycin (1)**

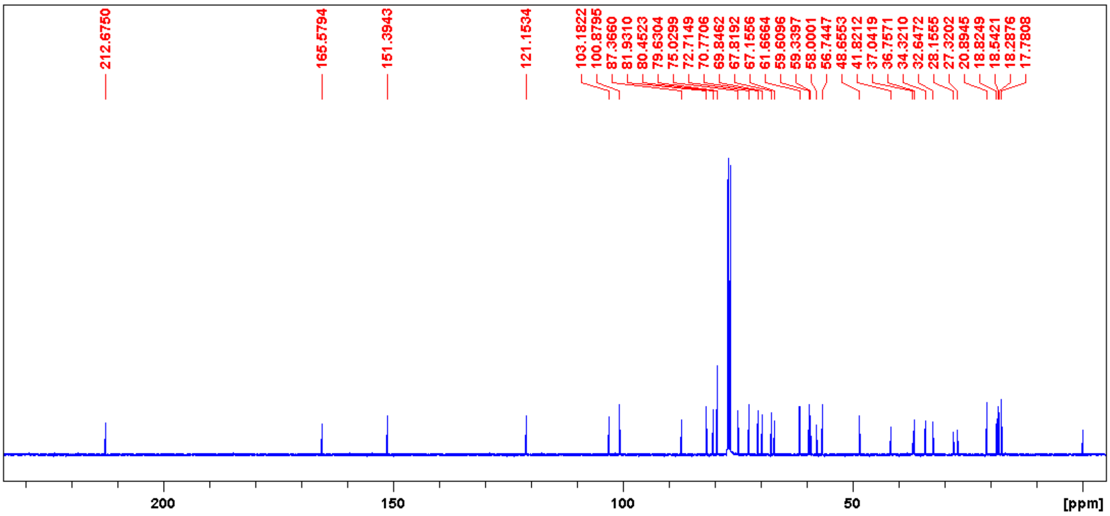

Figure S2. ^{13}C **NMR (100 MHz, $CDCl_3$) spectrum for dihydrochalcomycin (1)**

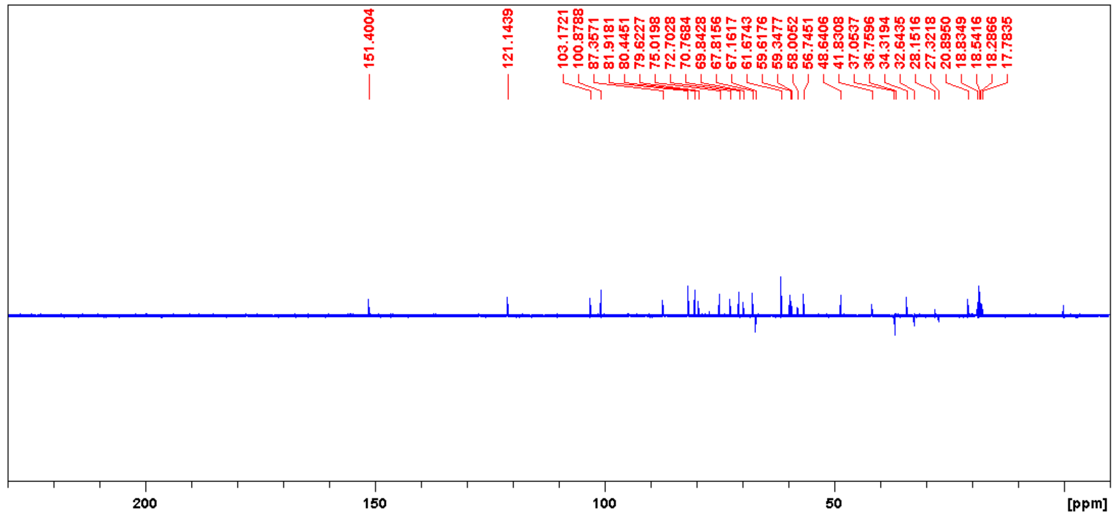

Figure S3. DEPT135 (100 MHz, $CDCl_3$) spectrum for dihydrochalcomycin (1)

4. The 1D NMR spectra for chalcomycin (2)

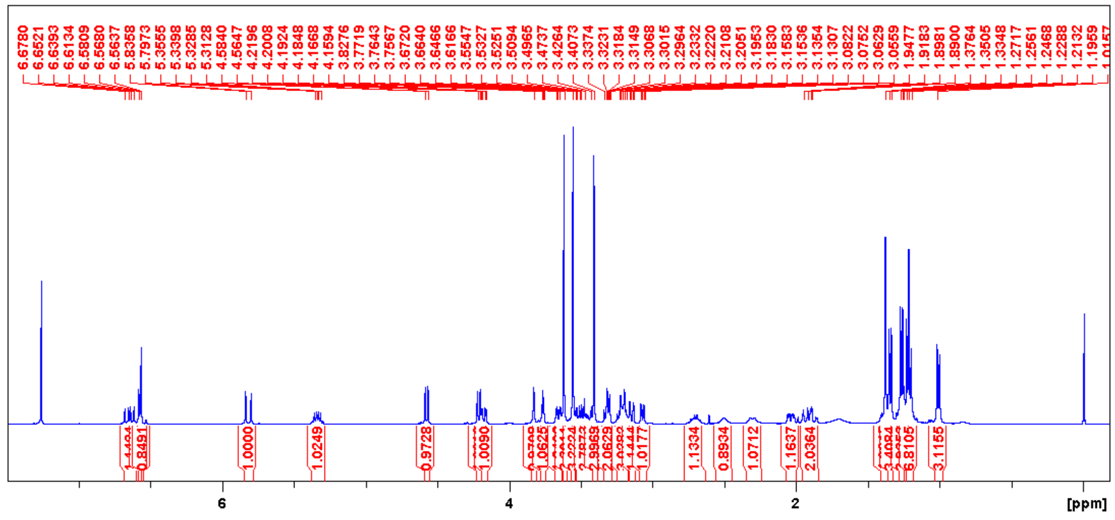

Figure S4. ^1H NMR (400 MHz, $CDCl_3$) spectrum for chalcomycin (2)

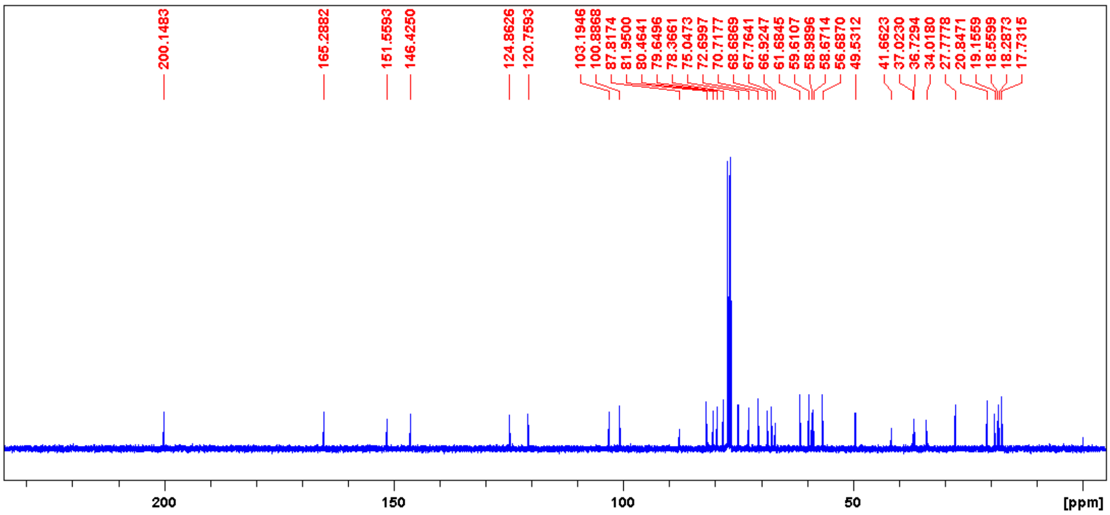

Figure S5. ^{13}C NMR (100 MHz, CDCl3) spectrum for chalcomycin (2)

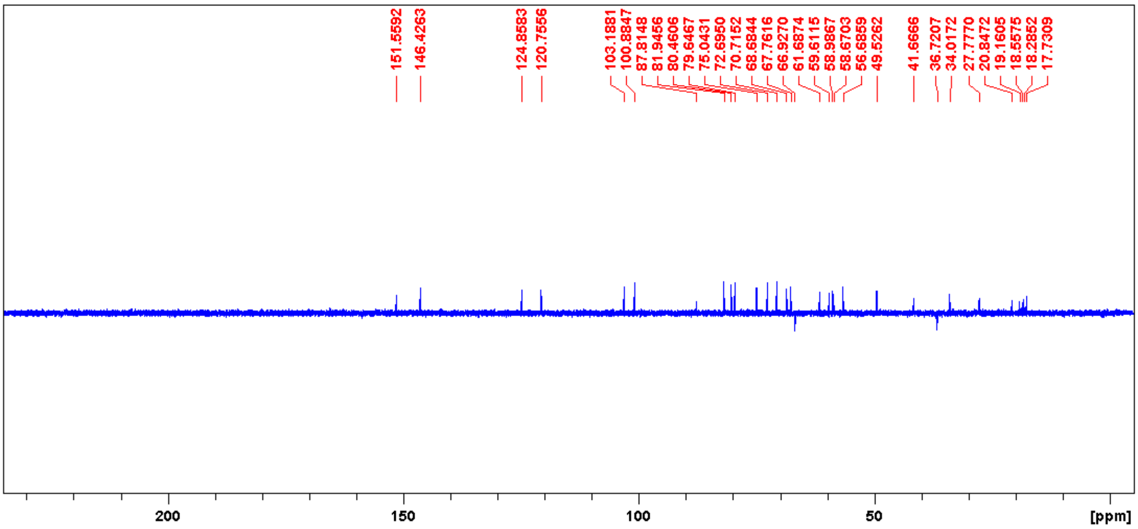

Figure S6. DEPT135 (100 MHz, CDCl3) spectrum for chalcomycin (2)

5. The 1D and 2D NMR spectra for chalcomycin E (3)

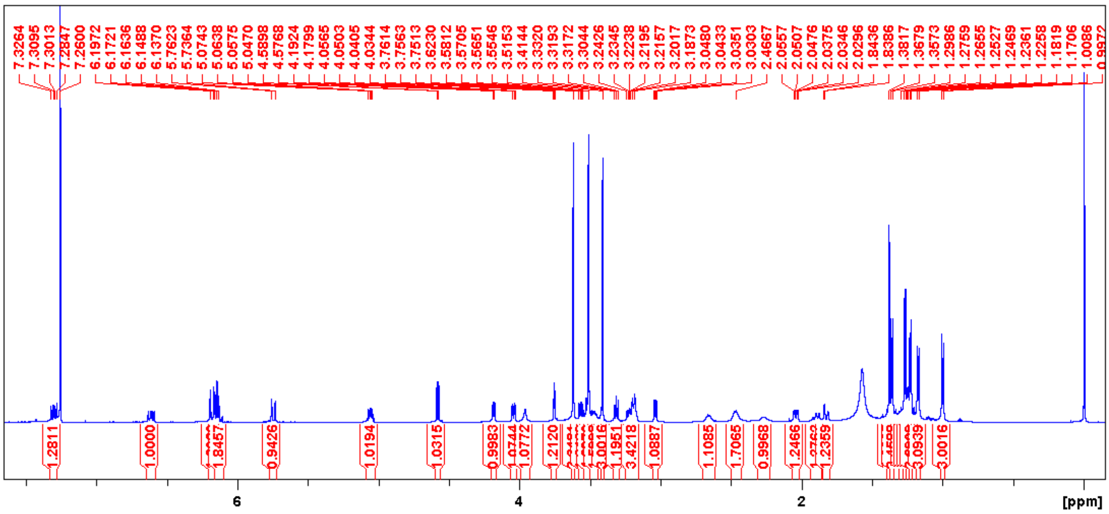

Figure S7. ^{1}**H NMR (600 MHz, CDCl**$_3$**) spectrum for chalcomycin E (3)**

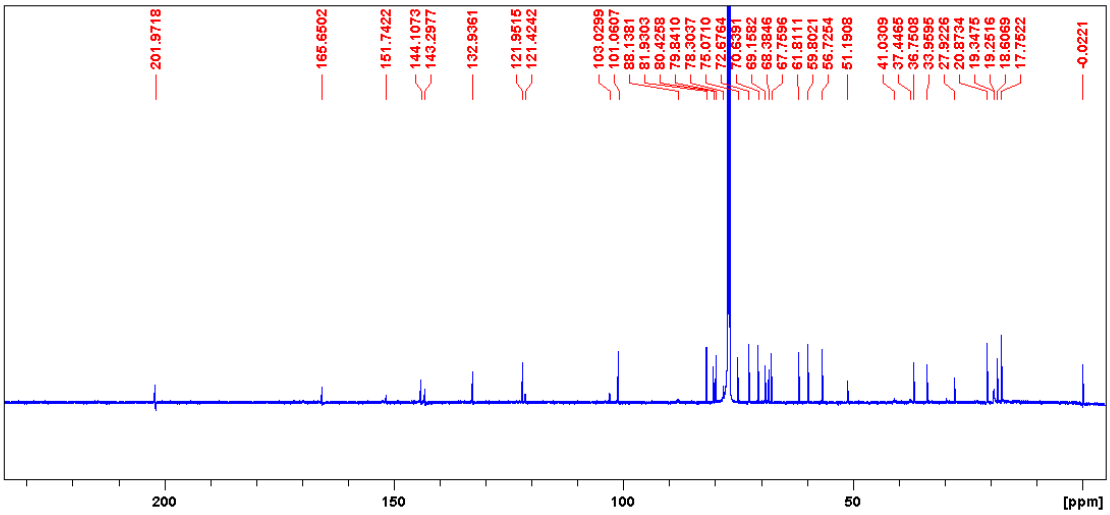

Figure S8. ^{13}**C NMR (150 MHz, CDCl**$_3$**) spectrum for chalcomycin E (3)**

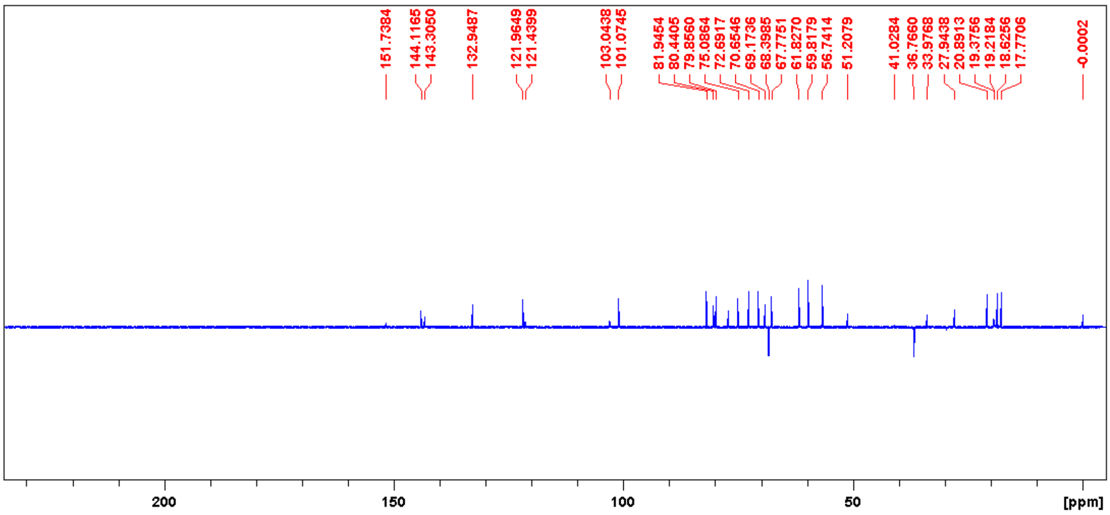

Figure S9. DEPT135 (150 MHz, $CDCl_3$) spectrum for chalcomycin E (3)

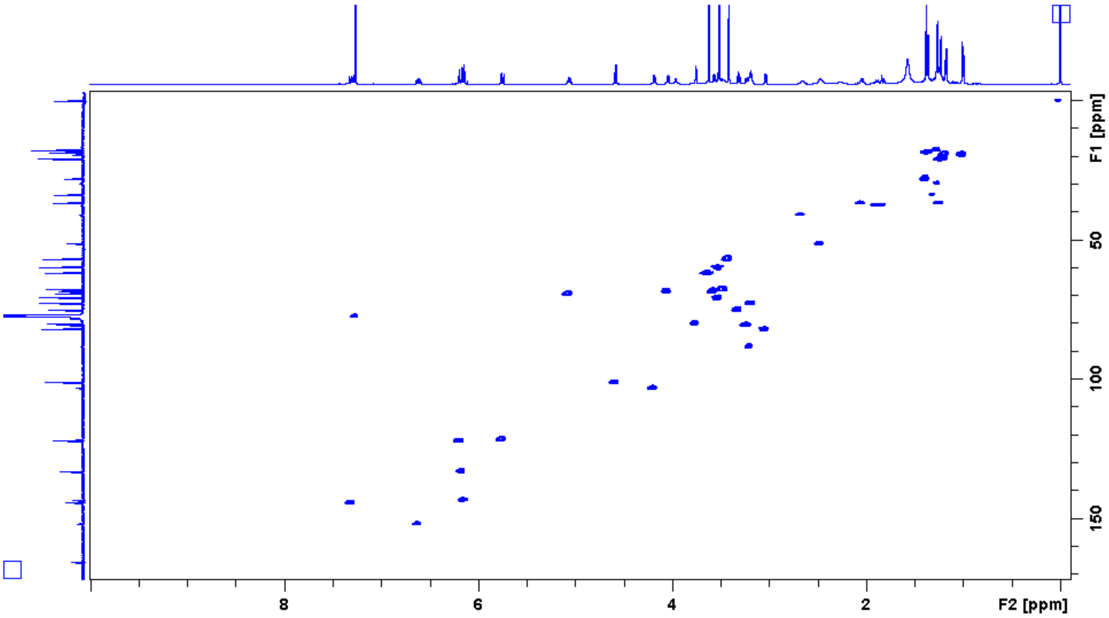

Figure S10. HSQC ($CDCl_3$) spectrum for chalcomycin E (3)

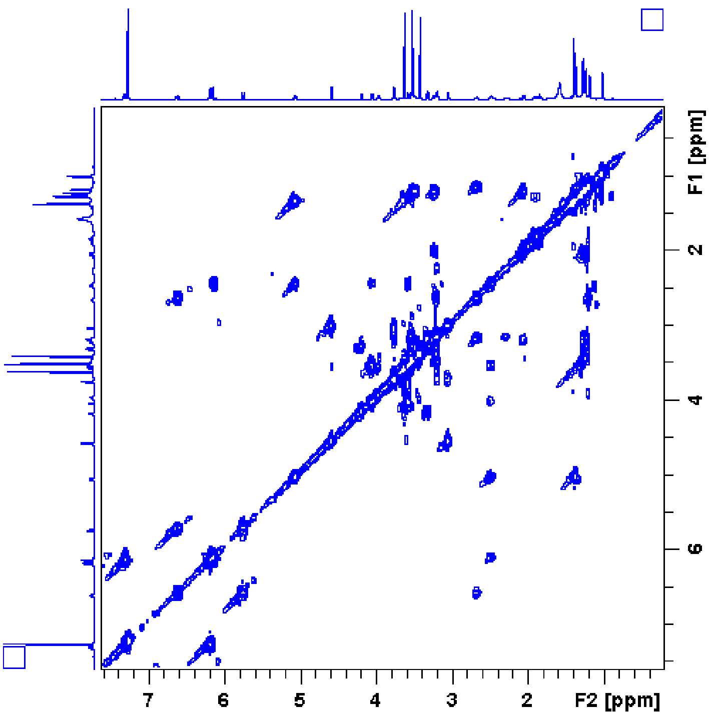

Figure S11. **^{1}H-^{1}H COSY (CDCl$_3$) spectrum for chalcomycin E (3)**

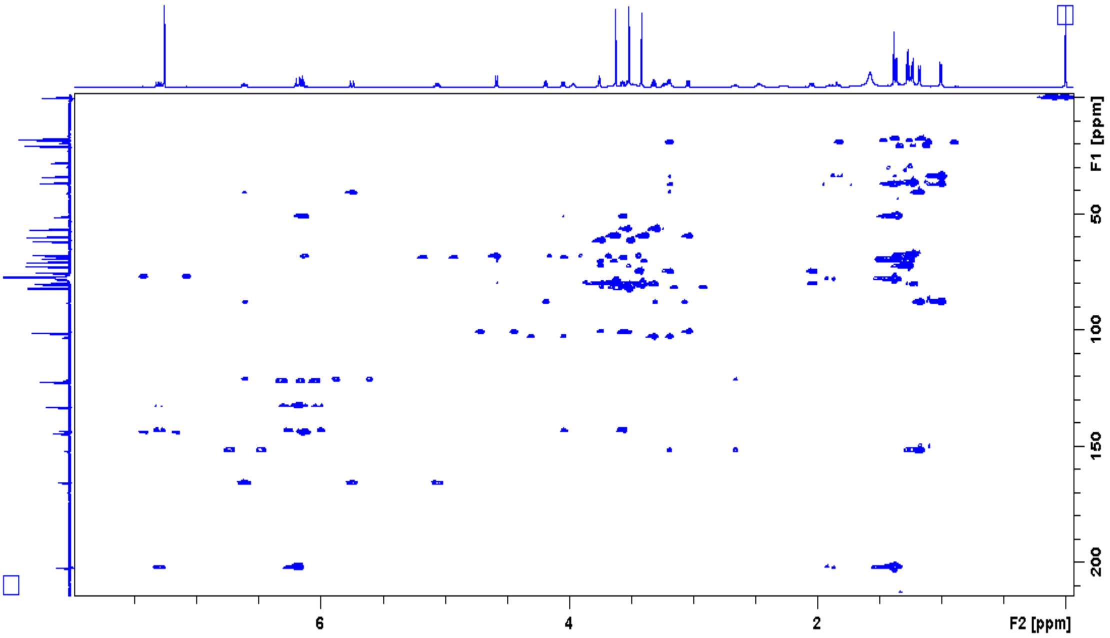

Figure S12. HMBC (CDCl$_3$) spectrum for chalcomycin E (3)

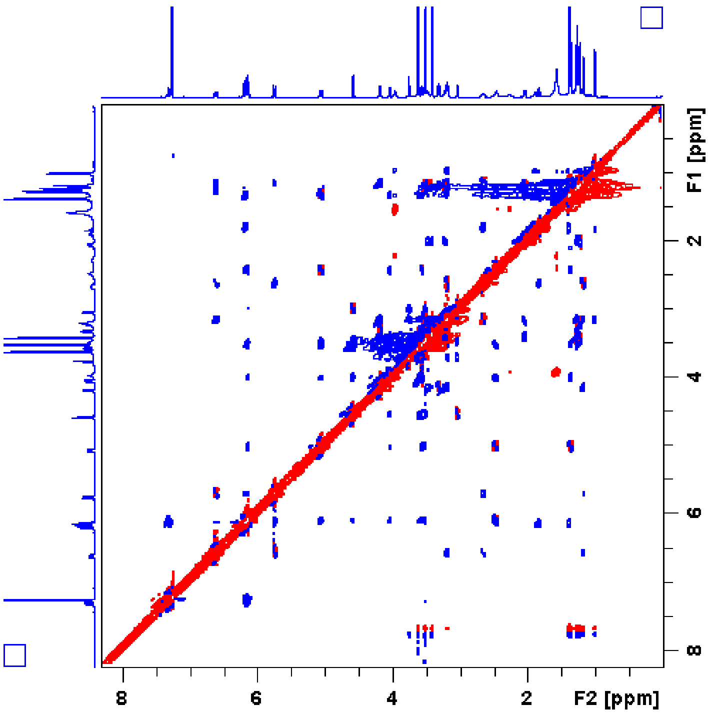

Figure S13. ROESY ($CDCl_3$) spectrum for chalcomycin E (3)

6. The UV spectrum for chalcomycin E (3)

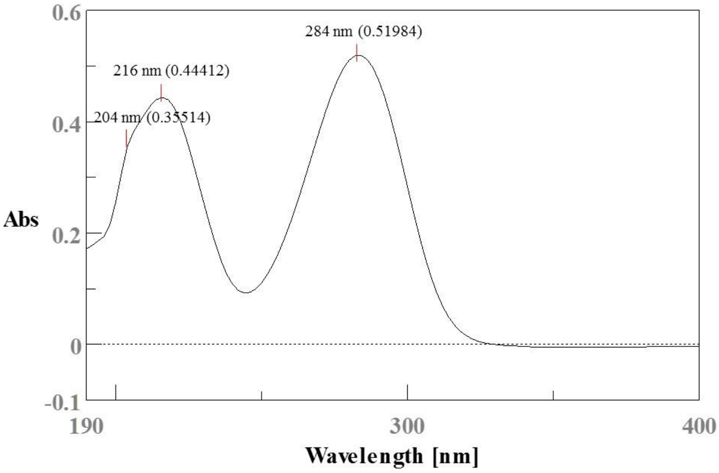

Figure S14. UV spectrum for chalcomycin E (3)

7. The HRESIMS spectrum for chalcomycin E (3)

Elemental Composition Report **Page 1**

Single Mass Analysis
Tolerance = 5.0 mDa / DBE: min = -1.5, max = 50.0
Element prediction: Off
Number of isotope peaks used for i-FIT = 3

Monoisotopic Mass, Even Electron Ions
459 formula(e) evaluated with 4 results within limits (up to 20 best isotopic matches for each mass)
Elements Used:
C: 0-100 H: 0-200 O: 0-100 Na: 0-1
AL-533
2016070408 150 (1.212) 1: TOF MS ES+ 6.82e+004

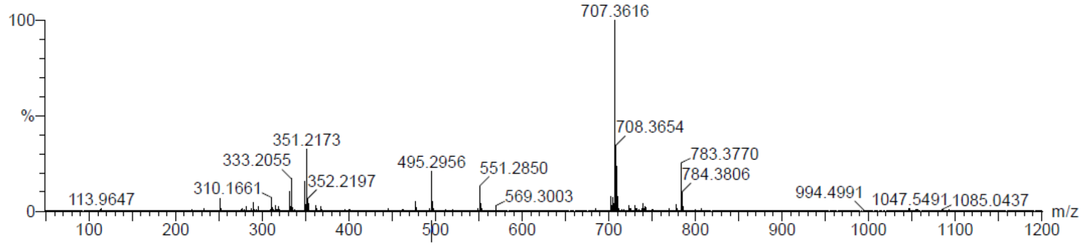

Minimum:				-1.5				
Maximum:		5.0	10.0	50.0				
Mass	Calc. Mass	mDa	PPM	DBE	i-FIT	Norm	Conf(%)	Formula
707.3616	707.3619	-0.3	-0.4	7.5	180.8	1.289	27.54	C35 H56 O13 Na
	707.3654	-3.8	-5.4	29.5	181.2	1.681	18.62	C53 H48 Na
	707.3643	-2.7	-3.8	10.5	180.6	1.110	32.95	C37 H55 O13
	707.3584	3.2	4.5	19.5	181.1	1.566	20.89	C44 H51 O8

Figure S15. HRESIMS spectrum for chalcomycin E (3)

8. References

1. Asressu, K. H.; Tesema, T. K. Chemical and Antimicrobial Investigations on Essential Oil of Rosmarinus officinalis Leaves Grown in Ethiopia and Comparison with other Countries. *J. Appl. Pharm.* **2014**, *6*, 132-142. http://nebula.wsimg.com/6db7597c765bf27d569c1e358ed05004?AccessKeyId=4323AF8BFBC2D34AB0BD&disposition=0&alloworigin=1.
2. Hidaki, M.; Yoshida, M.; Ogawa, N.; Kodani, S. Isolation and Structural Determination of Makinolide B from *Streptomyces* sp. MK-19. *Biosci., Biotechnol., Biochem.* **2013**, *77*, 1964-1966. doi: 10.1271/bbb.130204.
3. Celaya, L.; Viturro, C.; Silva, L. R. Chemical Composition and Biological Prospects of Essential Oils and Extracts of Aphyllocladus spartioides Growing in Northwest Argentina. *Chem. Biodiversity.* **2017**, *14*, e1600227. doi: 10.1002/cbdv.201600227.
4. Wansi, J. D.; Chiozem, D. D.; Tcho, A. T.; Toze, F. A. A.; Devkota, K. P.; Ndjakou, B. L.; Wandji, J.; Sewald, N. Antimicrobial and antioxidant effects of phenolic constituents from Klainedoxa gabonensis. *Pharm. Biol.* **2010**, *48*, 1124-1129. doi: 10.3109/13880200903486644.
